# Gibberellin induced transcription factor *bZIP53* regulates *CesA1* expression in maize kernels

**DOI:** 10.1371/journal.pone.0244591

**Published:** 2021-03-17

**Authors:** Huayang Lv, Xiao Li, Hui Li, Yufeng Hu, Hanmei Liu, Shengjuan Wen, Yangping Li, Yinghong Liu, Huanhuan Huang, Guowu Yu, Yubi Huang, Junjie Zhang

**Affiliations:** 1 College of Life Science, Sichuan Agricultural University, Yaan, Sichuan, China; 2 College of Agronomy, Sichuan Agricultural University/Key Laboratory of Crop Genetic Resources and Improvement, Chengdu, Sichuan, China; 3 Maize Research Institute, Sichuan Agricultural University/Key Laboratory of Crop Genetic Resources and Improvement, Ministry of Education, Chengdu, Sichuan, China; ICAR-Indian Institute of Agricultural Biotechnology, INDIA

## Abstract

Proper development of the maize kernel is of great significance for high and stable maize yield to ensure national food security. Gibberellin (GA), one of the hormones regulating plant growth, is involved in modulating the development of maize kernels. Cellulose, one of the main components of plant cells, is also regulated by gibberellin. The mechanism of hormone regulation during maize grain development is highly complicated, and reports on GA-mediated modulation of cellulose synthesis during maize grain development are rare. Our study revealed that during grain growth and development, the grain length and bulk density of GA-treated corn kernels improved significantly, and the cellulose content of grains increased, while seed coat thickness decreased. The transcription factor basic region/leucine zipper motif 53 (*bZIP53)*, which is strongly correlated with cellulose synthase gene 1 (*CesA1)* expression, was screened by transcriptome sequencing and the expression of the cellulose synthase gene in maize grain development after GA treatment was determined. It was found that *bZIP53* expression significantly promoted the expression of *CesA1*. Further, analysis of the transcription factor *bZIP53* determined that the gene-encoded protein was localized in the cell and nuclear membranes, but the transcription factor *bZIP53* itself showed no transcriptional activation. Further studies are required to explore the interaction of *bZIP53* with *CesA1*.

## Introduction

Corn is an essential agricultural food crop in China, which is the highest producer of corn in the world. Eighty-six percent of the maize produced is used in feed for pigs, chickens, cows, and beef cattle located in 85% of developed countries. With the development of agricultural livestock production, the status of corn as a feed crop in China is becoming increasingly important. Maize is historically known as a long-life food, rich in nutrients, such as protein, fat, vitamins, trace elements, and cellulose, and possesses great potential for the development of highly nutritional and bio-functional food. There are still many shortcomings in corn cultivation technology that affects corn growth and production. It is important to improve both the quantity and quality of production to achieve a high and stable yield of corn. Corn are the primary crop, and very significant to agricultural production. Which’s kernels quality is also directly related to food safety.

The use of scientific and technological methods to research and improve the development of corn is essential for increasing corn yield. Hormones regulate corn grain formation and growth at different stages of development, which affect the accumulation of various components in kernels. After soaking corn grains with gibberellin (GA), the 100-grain weight and grain yield of corn in field planting increased by 6.0% and 9.7%, respectively (P < 0.05) [1]. The germination percentage, α-amylase activity, and abortion rate of corn grains treated with 20 mg/LGA decreased, whereas the fresh and dry weight of the grains increased [2]. Spraying different concentrations of GA on corn ears increased the yield of corn by 0.84−25.14% [3].

Corn grains are composed of a seed coat, embryo, and endosperm. The seed coat is the outermost layer and primarily protects the grain. The embryo accounts for 80% of the weight of the grain and provides nutrients for the germination of the grain within the seed coat. The endosperm, as the most important structural part of the grain, is located at the innermost part of the grain and accounts for 10−15% of the weight. The seed coat is an integral part of the structure of maize kernels, and functions to protect seeds from mechanical damage and invasion by pests and diseases. The main components of the seed coat are cellulose, starch, and protein, and its thickness depends on the quantity of cellulose. The effect of cellulose on the flexibility of the seed coat has been studied [4].

In recent decades, research on cellulose has been a focus of modern biological foundations and agricultural production research centers. Cellulose synthase genes from a variety of sources have been successfully cloned and expressed in different systems. The plant cell wall is primarily composed of cellulose, which is formed during the growth process of plant cells and accounts for 10−14% and 40−60% of the primary and secondary cell wall dry weights, respectively—it is one of the most abundant polymers in nature [5]. The cellulose synthase complex located on the cell membrane, as the primary site of cellulose synthesis, is assembled in the Golgi apparatus, transported by secretory vesicles, and bound to the cells [6]. If any cellulose synthetic protein is missing from the cellulose biosynthesis complex, it will directly affect its biosynthesis. The synthesis of cellulose depends on the family of the cellulose synthase gene (*CesA*), which is a member of the polygene family in plants. At present, the *CesA* gene has been identified in many plants, and the CESA protein of all higher plants can be divided into six categories [7]. In *Arabidopsis thaliana*, the size of the *CesA* gene is 3.5 kb and contains 9−13 introns [8]. *A*. *thaliana* is a model plant for research, and the functions of its cellulose synthase members have been largely documented [9–16].

GA is one of the six common plant hormones, which has an efficient, stable, and safe effect on crop growth. GA can significantly promote the growth of stems and leaves, preserve flowers and fruits, and enhance fruit expansion. Previous studies have found that there are many different types of GA, but not all are bioactive. GA can play a role at different periods of plant growth, and also has various degrees of influence on cellulose synthesis. For example, muskmelons, treated with GA, showed an increase in the longitudinal diameter and weight of fruit, and yield per plant [17]. After spraying light sweet red cherries with GA, the longitudinal diameter, weight, and the size of pulp cells of fruits increased [18]. After the cotton was treated with exogenous GA, the cotton fiber exhibited thicker cell walls, length and dry weight were significantly increased [19]. From the examination of cross-sections of GA-treated Poplar tissue, the thickening of secondary cell walls, and an increase in cellulose content in the xylem was detected [20]. GA promotes cellulose synthesis by weakening the interaction of SLR1-NAC29/31 in rice [21]. During the development of Sorghum, Assessment of GAs in dwf1-1 revealed ablation of GA. GA ablation was antagonistic to the expression of three specific cellulose synthase genes resulting in cellulose deficiency and growth dwarfism, which were complemented by exogenous bioactive gibberellic acid application. [22]. Analysis of transgenic lines showed that cellulose synthase gene 1 (*CESA1*) could partially rescue irx1(*cesa8*) null mutants, resulting in complementation of the plant growth defect, collapsed xylem and cellulose content deficiency [23]. Most reports regarding the influence of GA on cellulose synthesis on crop growth and development are related to plant stems and leaves. There are few studies on the mechanism of the GA regulation of cellulose synthesis in corn grain growth and development.

In previous research work, we found that GA could affect the expression of some of the cellulose synthase enzymes involved in the process of corn grain development. GA-treated corn grains were analyzed by RNA-Seq, and the transcription factors (TF) regulating the expression of cellulose synthase were screened during grain development. It was found that the basic region/leucine zipper motif 53 (*bZIP53)* had a significant promotional effect on the expression of *CesA1*. The molecular regulation mechanism of TFs involved in corn cellulose synthesis was studied via molecular approaches, which laid a foundation for the study of the effect of GA on cellulose synthesis during corn grain development.

## Materials and methods

### Experimental materials

In this study, the maize inbred line Mo17 was used as the plant material. During March–May 2017 and March–May 2018, the corn seeds were planted in batches at the Chongzhou Farm of the Sichuan Agricultural University in China, using their field management techniques. Maize seeds taken 5 days after pollination (DAP), 7 DAP, 10 DAP, 15 DAP, 20 DAP, 25 DAP, and 30 DAP, were used for the determination of grain cellulose content and sectioning. The roots, stems, and leaves of the six-leaf stage of maize, the filaments and pollen of the flowering stage, and the seeds, embryos, and endosperm of 15 DAP were used for semi-quantitative experiments. All materials were frozen with liquid nitrogen immediately after sampling and stored at −70°C [24, 25]. The leaves from maize plants with two leaves and one heart were used for subcellular localization experiments. The 10 DAP seeds were treated with 50 mM GA and incubated in a shaker incubator at 28°C, and 6.28 rad/s Seeds at 10 DAP were used for transient expression. Each set of experiments was replicated three times.

### Investigation of maize seed morphological characteristics

The indexes of ear length, ear diameter, shaft thickness, grain width, grain length, grain thickness, 100-grain weight, and bulk density of maize seeds were measured.

### Determination of cellulose content in maize seeds

The grains from the GA treatments at different times were used to determine the change in the cellulose content that was determined by the method of Van Soest [26].

### Observation of cellulose content and cell wall thickness

The seeds treated with GA for different times were sectioned and stained for microscopy using the Crocus O-solid green solution, plant tissue staining method [27].

### RNA extraction and reverse transcription

The total RNA of each sample was extracted with the TRIzol reagent (Invitrogen, China) and digested with DNase. A 1.5 ng sample of total RNA was reverse transcribed into cDNA using the Reverse Transcription Kit, Prime ScriptTM RT (Takara, Dalian, China.)

### RNA-Seq data analysis

According to RNA-Seq analysis of maize Mo17 kernels sprayed with GA, the genes with FDR ≤ 0.01, log2Ratio / ≥ 1, and expression > 0 were set up to screen the differentially expressed genes, and the TFs and target genes regulated by GA in this experiment were selected.

### Gene cloning and sequence analysis

**Fig 1:**
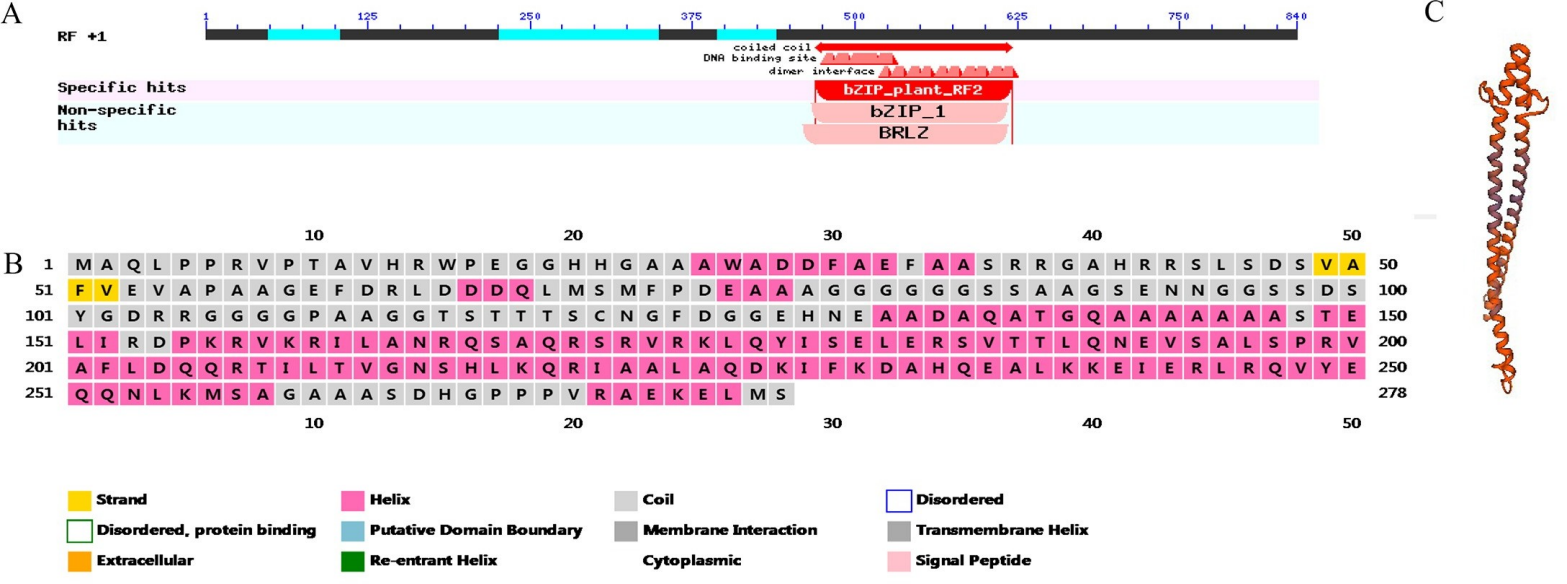
(A)Transcription factor *bZIP53* domain analysis (B) Secondary structure analysis of transcription factor *bZIP53* (C) Analysis of the tertiary structure of transcription factor *bZIP53*.

According to the gene sequence of *bZIP53* in the maize reference genome sequence published on the Gramene (http://ensembl.gramene.org/genome_browser/index.html) website, specific primers were designed by Primer 5 software: 5′-ATGTGACCCCTCCAATTCCA-3′(forward) and 5′-CATCCTTGAAAATCTTGTCCTGA-3′ (reverse). The *bZIP53* cDNA was amplified by PCR using genomic DNA from 15 DAP seeds of Mo17 inbred line, and its conserved domain, secondary structure, and tertiary structure were analyzed, respectively, by NCBI BLAST (https://blast.ncbi.nlm.nih.gov/Blast.cgi) (Fig 1A), PSIPRED (http://bioinf.cs.ucl.acuk/psipred/) (Fig 1B), and SWISS-MODEL (http://swissmodel.expasy.org/) (Fig 1C.)

According to the promoter sequence of the *CesA1* gene in the maize reference genome sequence published on the Gramene website, the specific primers were designed by Primer 5 software: 5′-GTGAACTGCCCACCTCCA-3′(forward) and 5′-CTCCGCTCCCTCCCC-3′ (reverse), and the promoter was PCR amplified from the DNA of the Mo17 inbred line. Promoter analysis of the *CesA1* gene was performed using Plant Care (http://bioinformatics.psb.ugent.be/webtools/plantcare/html/).

The promoter sequences of the *bZIP53* and *CesA1* genes and the ligation vectors *bZIP53*-007 and *CesA1*-007 of the pGlone007 Blunt Simple Vector Kit were separately constructed to facilitate subsequent construction of expression vectors required for various experiments by recombinant methods.

### Analysis of *bZIP53* expression

To quantify the expression of *bZIP53* in grains after GA treatment via real-time PCR technique, following primers using Primer 5 software: 5′- GCTGTCTCCCCGTGTGGC-3′ (forward) and 5′-CCTCTTGATGAGCATCCTTGAAAC-3′ (reverse) To explore the expression of *bZIP53* in different maize tissues, ZmACTIN was used as an internal reference gene and was analyzed by semi-quantitative experiments [27].

### Subcellular localization

By recombinant methods, using *bZIP53*-007 as the amplification template, the recombinant primers designed by Primer 5 software: 5′-ATTTGGAGAGGACAGGGTACCATGGCGCAGCTGCCG-3′ (forward) and 5′-GGTACTAGTGTCGACTCTAGAGCTCATGAGCTCTTT-3′ (reverse), were used to connect *bZIP53* to p2300-35S. The fusion expression vector *bZIP53*-eGFP was constructed upstream of eGFP in the -eGFP vector, and the transformation of maize leaves was done [28]. This experiment was used to analyze the location of the protein encoded by *bZIP53* in the cell.

### Transient transformation experiment of maize endosperm

To investigate the effect of *bZIP53* on *CesA1* expression, we constructed the transient transformation vectors of *bZIP53* and *CesA1* promoters. Using the recombinant method, *bZIP53*-007 was used as an amplification template, and the Primer 5 software was used to design recombinant Primer: 5′-ACGGGGGACTCTAGAGGATCCATGTGACCCCTCCAATTCCA-3′ (forward) and 5′-CGATCGGGGAAATTCGAGCTCCATCCTTGAAAATCTTGTCCTGA-3′ (reverse) for amplification of recombinant fragments. Using *CesA1*-007 as the amplification template, recombinant primers were designed with the Primer 5 software: 5′GCCAAGCTTGCATGCCTGCAGGTGAACTGCCCACCTCCA-3′ (forward) and 5′-CGGACCTTTGCACTTGGATCCCTCCGCTCCCTCCCC-3′ for the (reverse) for amplification of recombinant fragments. The pBI221 vector was double digested with BamH I and Pst I enzymes at 37°C for 4 h (the LUC reporter gene was used to replace the GUS reporter gene upstream of pBI221, the constitutive promoter 35S was replaced, and the first intron of the maize AdhI gene was inserted to enhance promoter activity.) Ligation of the fragments with DNA ligase I was conducted to recover the purified recombinant fragments and double digested pBI221 vector construction, bands. Then, the maize transformation for transient expression of transgene was conducted according to the method of Hu [29]. The specific operation was reported by Xiao [30].

### Identification of the binding region of *bZIP53* and *CesA1*

Twenty-four cis-acting elements (S1 Table) were predicted in the *CesA1* promoter, and the elements of TC rich, OBP1, and I Box were deleted one by one. The recombinant primers were designed with the Primer 5 software: *CesA1*:Mu1-F:GCCAAGCTTGCATGCCTGCAGCAACCCCTCCTTAGTTTTAGGAATA *CesA1*:Mu2-F:GCCAAGCTTGCATGCCTGCAGAAGCAAGGAGAAATATGCATGTACT *CesA1*:Mu3-F:GCCAAGCTTGCATGCCTGCAGAAAAGGAAAAAAACAAATAATCAG

The results were named *CesA1*:Mu1, *CesA1*:Mu2, and *CesA1*:Mu3. The Maize endosperm was bombarded with gene gun for transient expression to verify the effects of *bZIP53* on the activity of the promoter after deletion.

### Transcriptional activation

Recombinant methods, using *bZIP53*-007 as the amplification template and recombinant primers were designed by the Primer 5 software: 5′-TCAGAGGAGGACCTGCATATGATGTGACCCCTCCAATTCCA-3′ (forward) and 5′-CCGCTGCAGGTCGACGGATCCCATCCTTGAAAATCTTGTCCTGA -3′ (reverse). *bZIP53* was ligated to the pGBKT7 vector and named *bZIP53*-pGBKT7. The fusion expression vector *bZIP53*-pGBKT7 was used for transforming yeast strain AH109 by LiAc-PEG transformation method and was selected from SD/-Trp and SD/-Trp-His-Ura solid-deficient medium containing X-α-gal. The cells were cultured at 28°C for 3 d, and it was determined whether the TF *bZIP53* itself had transcriptional activity.

### Interaction between transcription factor *bZIP53* and *CesA1*

The recombinant method used *bZIP53*-007 as the amplification template and recombinant primers were designed using Primer 5 software: 5′-GTACCAGATTACGCTCATATGATGTGACCCCTCCAATTCCA-3′ (forward) and 5′-CAGCTCGAGCTCGATGGATCCCATCCTTGAAAATCTTGTCCTGA-3′ (reverse). *bZIP53* was ligated to pGADT7-Rec2 to construct the fusion expression vector *bZIP53*-pGADT7-Rec2. The recombinant method used *CesA1*-007 as the amplification template and recombinant primers were designed using Primer 5 software: 5′-GACTCACTATAGGGCGAATTCGTGAACTGCCCACCTCCA-3′ (forward) and 5′-CGGATCGATTCGCGAACGCGTCTCCGCTCCCTCCCC-3′ (reverse). The fusion expression vector *CesA1*-His2 was constructed by ligating the *CesA1* gene promoter fragment to the His2 vector [30]. The yeast expression vector was co-transformed into the yeast Y187 strain using the LiAc-PEG method, and the interaction between *bZIP53* and the *CesA1* promoter was observed.

## Results

### RNA-Seq data analysis

According to RNA-Seq analysis of maize Mo17 kernels sprayed with GA, the genes with FDR ≤ 0.01, log2Ratio / ≥ 1, and expression > 0 were selected as the differentially expressed genes. A total of 917 differentially expressed genes were obtained, of which 562 were upregulated, and 355 were downregulated. There were 20 families of 52 commented TFs (S2 Table), and 13 target genes were screened, of which four were cellulose synthase genes, accounting for 44.4% of maize cellulose synthase (S3 Table). It was speculated that GA treatment had a regulatory effect on cellulose synthase in corn kernels, and the TFs were further screened by expression level to determine the GA regulation of cellulose synthase in maize kernels.

The co-correlation analysis of the expression of four corn cellulose synthase genes in different tissues and grains of maize was conducted using the existing *bZIP53* gene in the database and the expression of four corn cellulose synthase genes in different tissues and grains of maize (S4 Table). *BZIP53* exhibited a higher correlation with the *CesA1* gene in corn grains, and the correlation coefficient was above 0.8, but was lower than that in other parts, indicating that *bZIP53* had a similar gene expression pattern as *CesA1*.

#### Determination of cellulose content of maize seeds

**Fig 2:**
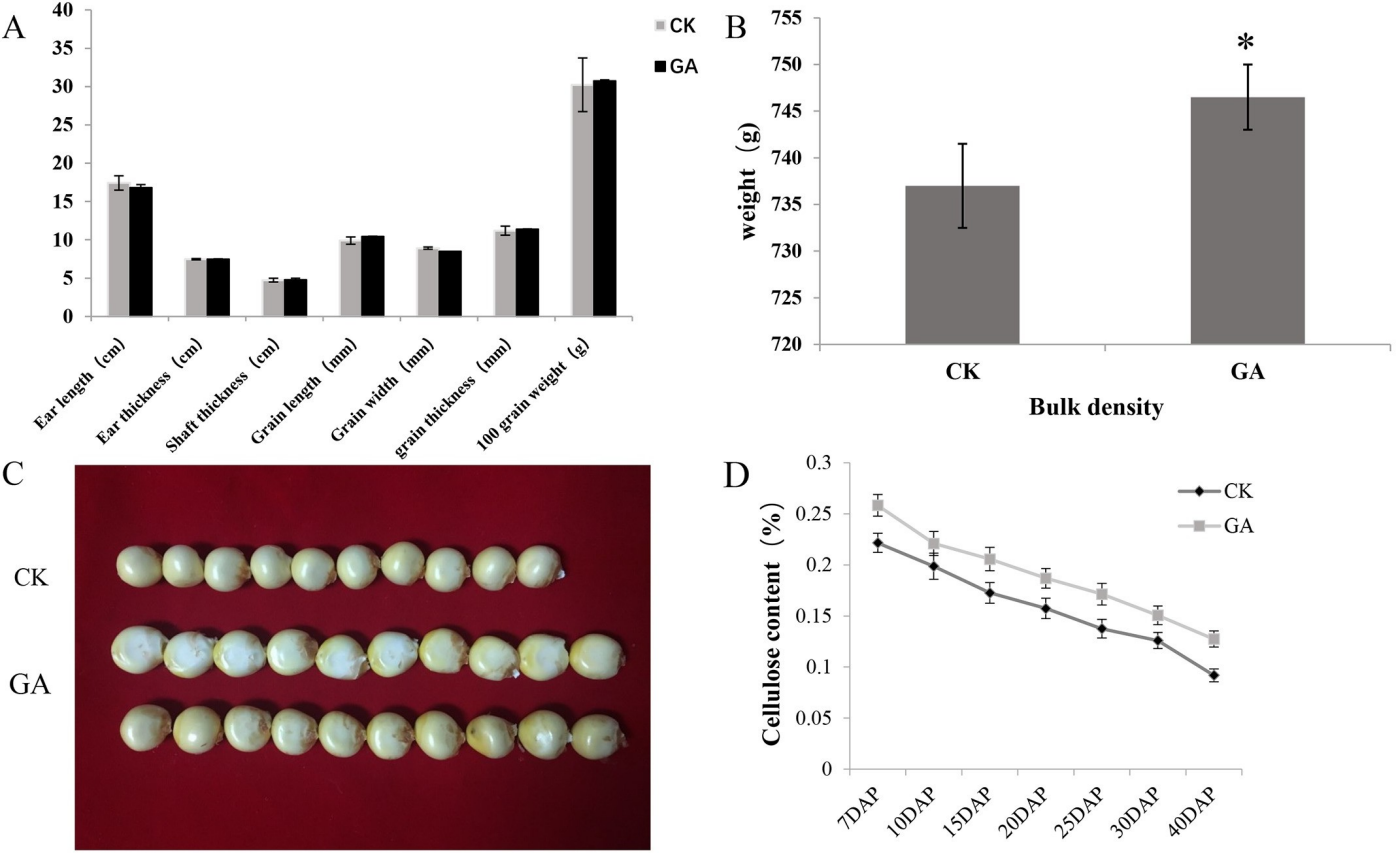
Effect of GA on corn yield traits, kernel size, and cellulose content. CK: Untreated (control), GA: Gibberellin treatment. (A)The indicators ear length, ear diameter, shaft thickness, grain length, grain width, grain thickness, and 100-grain weight. (B)Bulk density (average of three independent experiments.) (C) Comparison of GA-treated and untreated (control) corn kernels. (D) Cellulose content of GA-treated and untreated corn grains at different stages. Three independent experiments * p<0.05, ** p<0.01 significantly different from control.

Thirty maize plants with similar growth were selected, and GA was used to treat the experimental group. Fifty mM GA was applied to the corn ear husks the day before pollination when the temperature was low and the sun weak. This procedure was continued daily until 30 DAP. Thirty maize plants were treated with sterile water as the control group, and the field experiment was conducted for two consecutive years. There were significant differences in corn grains at harvest. The indicators ear length, ear thickness, shaft thickness, grain width, grain length, grain thickness, 100-grain weight, and bulk density were measured. It was found that 100-grain weight, grain length, and bulk density increased significantly after treatment with GA (Fig 2A–2C). The cellulose content at 7 DAP, 10 DAP, 15 DAP, 20 DAP, 25 DAP, 30 DAP, and 40 DAP maize seeds (with root cap removed) was determined. The cellulose content was highest in the early stage of grain development and decreased with the growth of the maize seeds. However, at each stage of maize grain development, the cellulose content of GA-treated maize seeds was higher than that of the control group (Fig 2D), and it was confirmed that GA treatment increased the cellulose content of maize seeds.

### Observation of cellulose thickness and seed coat

**Fig 3:**
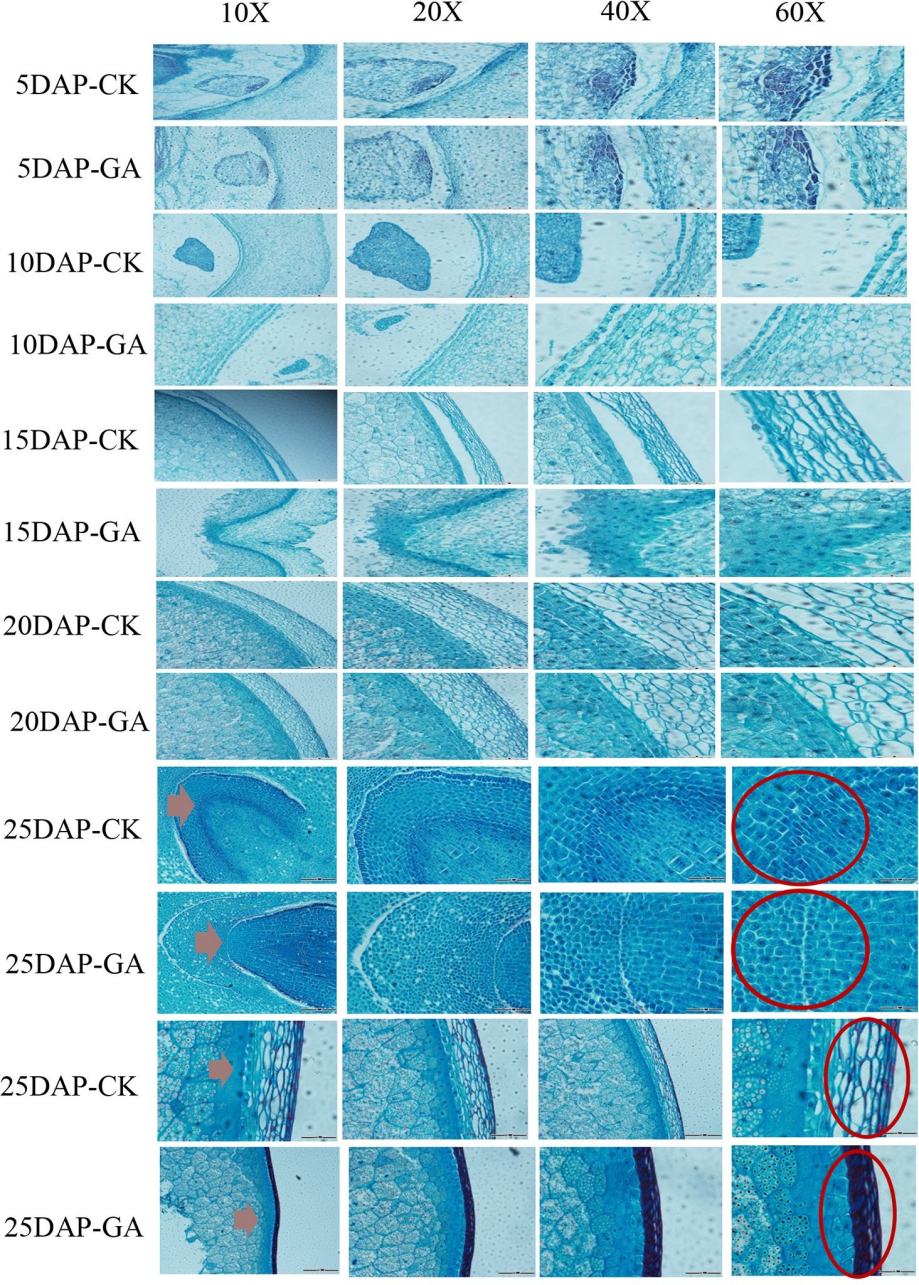
Cellulose thickening of 5–25 DAP maize seed sections at 10, 20, 40 and 60X magnification. CK: Untreated (control), GA: Gibberellin treatment. The red arrow shows the seed coat. (thinner in the GA treatment though with higher cellulose content, shown by the deeper color).

Seeds at 5 DAP, 10 DAP, 15 DAP, 20 DAP, and 25 DAP were used for tissue sectioning, and the changes in cellulose content in the grain and the effect of GA on grain development were observed. We observed the same part of the grain under 10X, 20X, 40X, and 60X magnification. The results are shown in Fig 3. As time elapsed, the shape of the maize seed coat cells treated with GA changed significantly, and the cell area and gap decreased. There were significant increases in growth rate, tissue enlargement, and boundary cell number compared to the control group. Simultaneously, staining revealed that the outermost cells of the GA-treated 25 DAP corn kernels had formed a flat structure, and showed a higher cellulose content and thinner seed coat than the control group (Fig 3). During the growth and development of grain, the seed coat cells of the grain thickened during the late stage of development. The GA-treated seeds exhibited an increase in cell wall thickening and the speed of thickening, and a thinner seed coat.

### Gene cloning and sequence analysis

Specific primers were designed according to the *bZIP53* gene sequence in the maize reference genome sequence published on the Maize GDB website. The coding sequence length of *bZIP53* was 976 bp according to PCR amplification with 15 DAP grain cDNA as a template after maize inbred line Mo17 pollination (S1 Fig). The *bZIP53* gene was ligated into the cloning vector pGlone007 Blunt Simple Vector Kit, positive clones were detected, and the sequence was analyzed by DNAMAN software after sequencing analysis. The alignment showed that the *bZIP53* gene of the positive clone and the amino acid sequence encoded by *bZIP53* in the reference genome were highly correlated.

According to the whole genome sequencing results of maize inbred lines, the amplification primers of the CesA1 promoter were designed, and the maize inbred line Mo17 DNA was used as a template for PCR amplification. The CesA1 promoter fragment and the vector of pGlone007 Blunt Simple Vector Kit were constructed, and the positive clones were selected. Sequencing analysis after detection showed that the cloned promoter sequence and the reference genome sequence differed at few sites.

### Expression analysis of *bZIP53* in various tissues of maize

**Fig 4:**
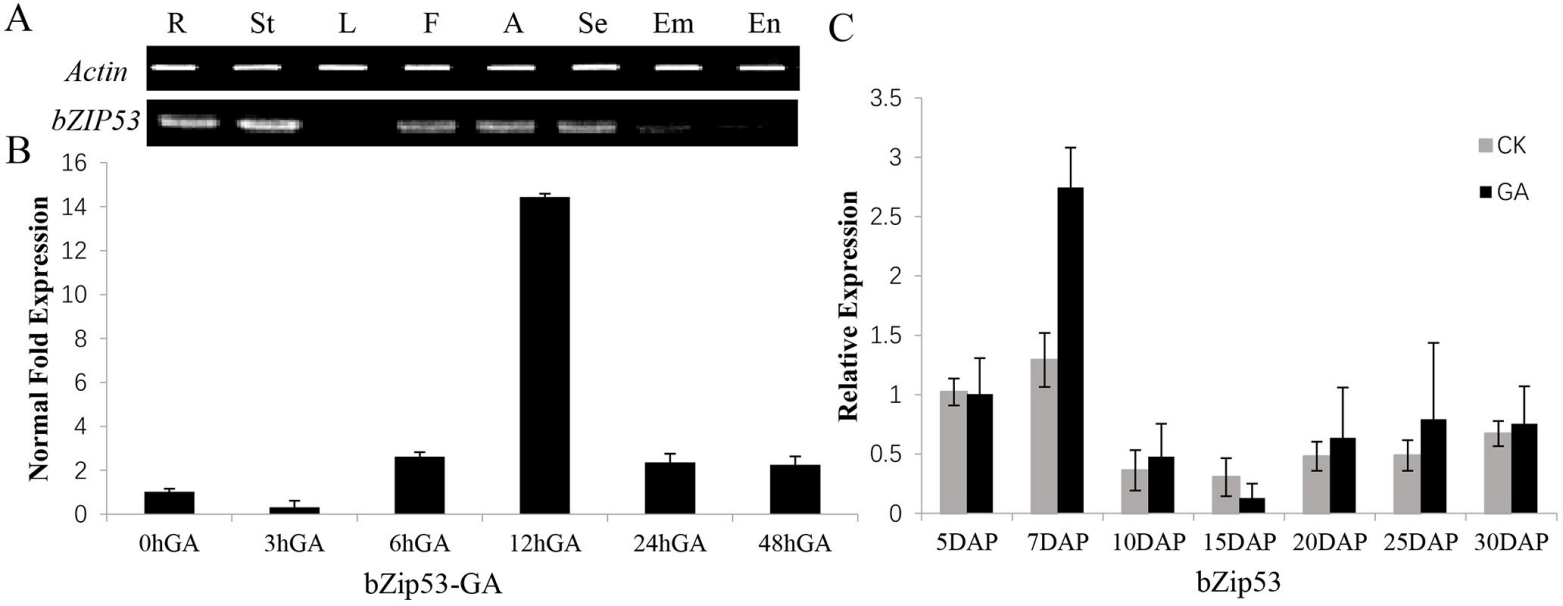
Semi-qRT and qrt-PCR based expression analysis of *bZIP53* in various maize tissues. CK: Untreated (control), GA: Gibberellin treatment. (A)Expression of *bZIP53* in various tissues of maize. R: root; St: stem; L: leaves; F: filaments; A: anthers, Se: seeds; Em: embryo; En: endosperm. (B)Expression of *bZIP53* at different times after GA treatment of maize seeds. (C)Expression of *bZIP53* at different times after GA treatment of maize leaves in the field. Three independent experiments. * p<0.05, ** p<0.01 significantly different from control.

Semi-quantitative RT-PCR analysis of *bZIP53* showed that the *bZIP53* gene was not expressed in leaves (Fig 4A) but was expressed in the embryos and endosperm of maize grains. A two-part experiment was performed to demonstrate the effects of the expression level of the TF *bZIP53* after GA treatment. The maize inbred line Mo17 was used for the treatment of 10 DAP seeds with 50 mM GA, and samples were taken at 3 h, 6 h, 12 h, and 48 h after GA treatment. The 0 h after GA treatment sample was taken as control and the experiment showed that the expression of TF *bZIP53* gene was affected by GA. The amount of expression was highest after the treatment of the grains for 12 h (Fig 4B).

Maize grain cDNA from the control group and from 5 DAP, 7 DAP, 10 DAP, 15 DAP, 20 DAP, 25 DAP, and 30 DAP with GA treatment were used as templates for real-time PCR, for analysis of the expression of the *bZIP53* gene in maize seeds after GA treatment. After treatment with the GA, the expression level of bZIP53 was highest at 7 DAP stage and the expression was higher at 20–30 DAP stages than control (Fig 4C).

### Subcellular localization of *bZIP53*-encoded protein

**Fig 5:**
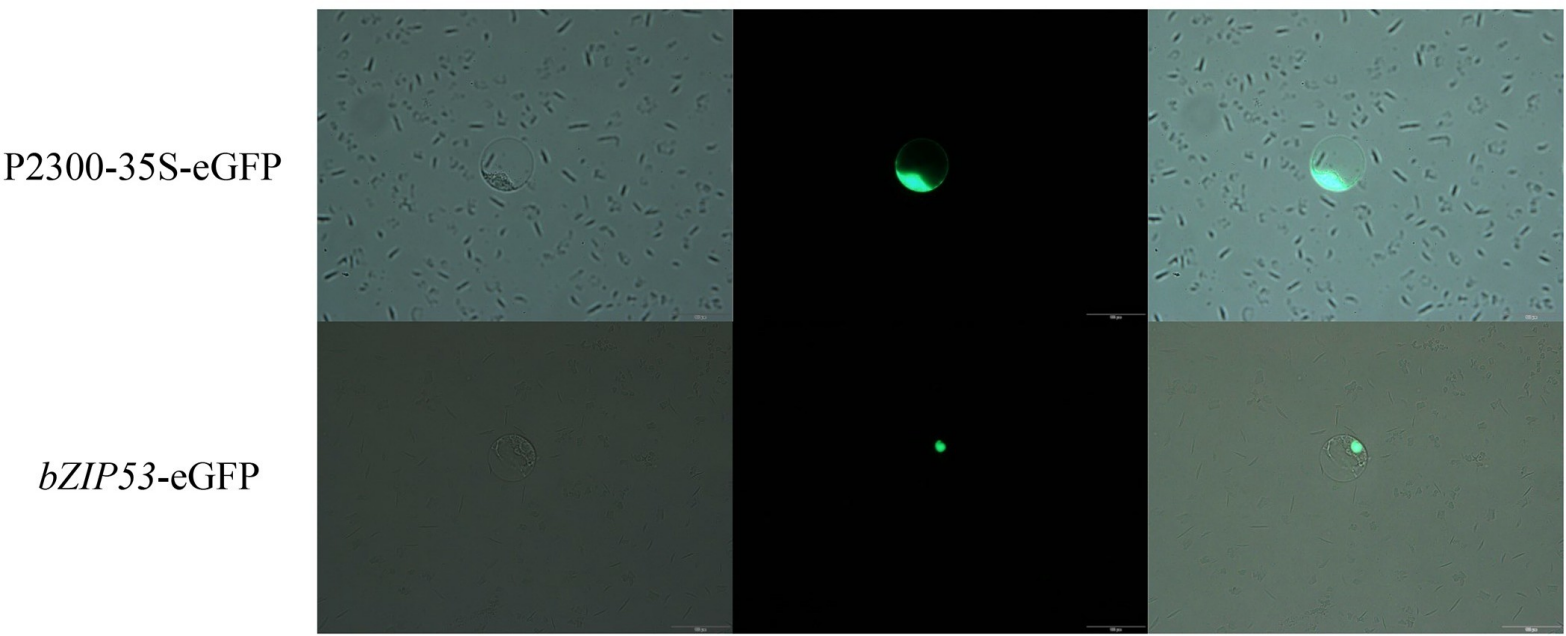
Identification of subcellular localization of *bZIP53*-encoded protein. Fluorescence microscopy analysis of the maize leaf protoplast cells expressing either GFP under control of 35S promoter or GFP fused with bZIP53 gene. (scale bar = 100 um).

To further determine the position of the *bZIP53*-encoded protein in the cell, the *bZIP53* gene was cloned into the expression vector p2300-35S- eGFP and transformed into maize leaf protoplast cells. It was observed that the protein encoded by p2300-35S-eGFP in the control group could produce green fluorescence in the cell membrane, cytoplasm, and nucleus. The protein encoded by *bZIP53*-eGFP showed green fluorescence in the nucleus. These results indicated that the protein encoded by the *bZIP53* gene was localized in the nucleus (Fig 5).

### Transformation of maize endosperm for transient expression

**Fig 6:**
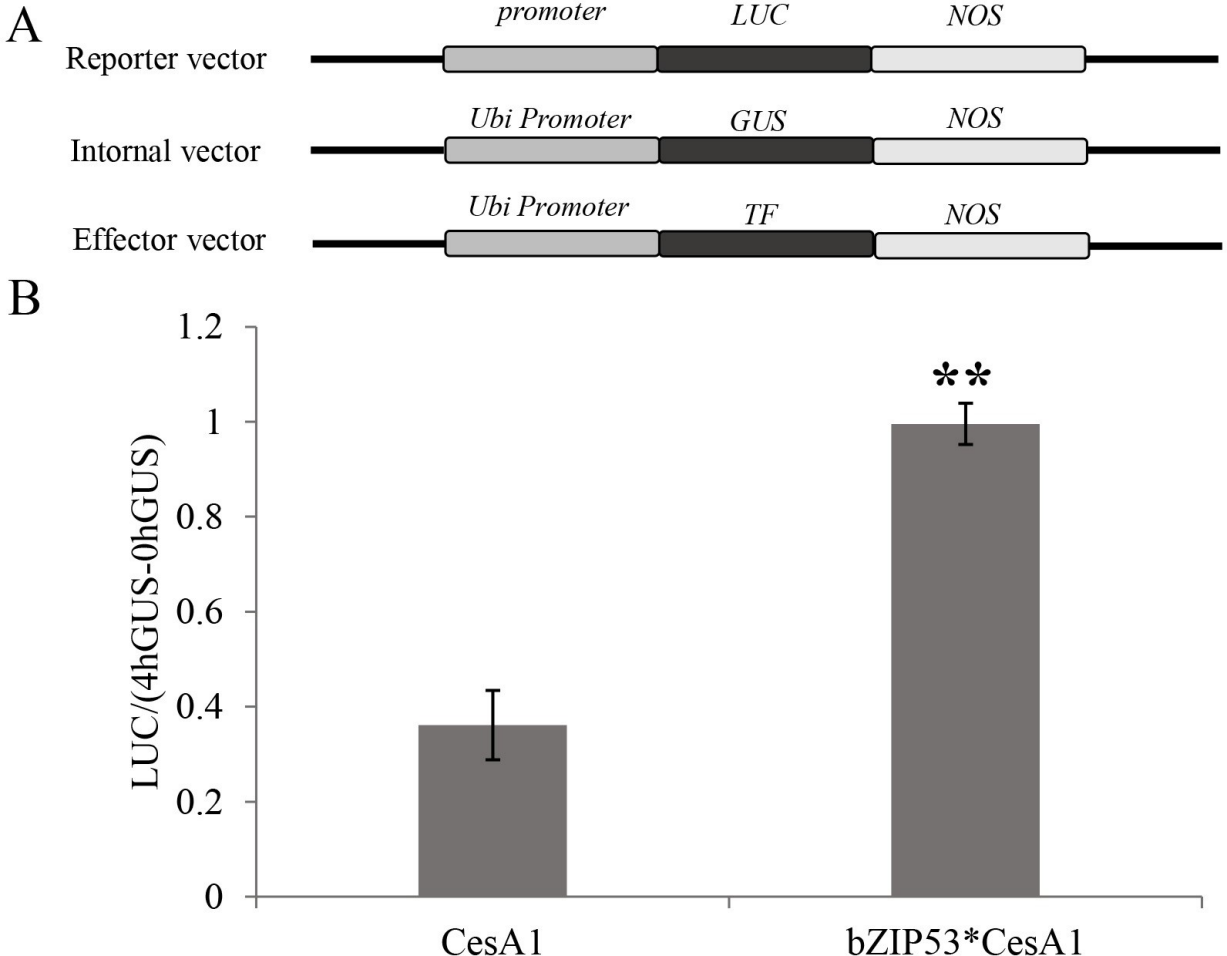
Transient assay for the interaction between *bZIP53* and promoters of *CesA1* gene in maize seeds. (A) Diagrammatic representation of constructs used in the study. CesA1 gene full-length promoter was cloned in reporter vector and bZIP53 gene was cloned into the effect vector, respectively. (B)The reporter construct and the internal control construct were co-bombarded into maize seeds either with or without the effector construct. LUC activity was normalized to GUS activity in every independent transformation. The data are given as the means±SD of three replicates.

Maize endosperm cells were transformed via gene gun to find out the effect of the overexpression of the TF *bZIP53* on the activity of the *CesA1* gene promoter in maize seeds. The effects of the TF *bZIP53* on the activity of the *CesA1* promoter were analyzed by comparing the difference in the ratio of LUC/GUS (4h−0h) after the co-bombardment of the CesA1 gene promoter with the TF” to “after the co-bombardment of the reporter construct harboring gene for LUC under control of CesA1 gene promoter with effector construct having bZIP53 TF gene. The effects of TFs on the activity of the cellulose synthase gene were analyzed by the ratio of LUC/(4hGUS−0hGUS). As shown in Fig 6, *bZIP53* overexpression significantly promoted the activity of the *CesA1* promoter.

#### Identification of the binding region of *bZIP53* and *CesA1*

**Fig 7:**
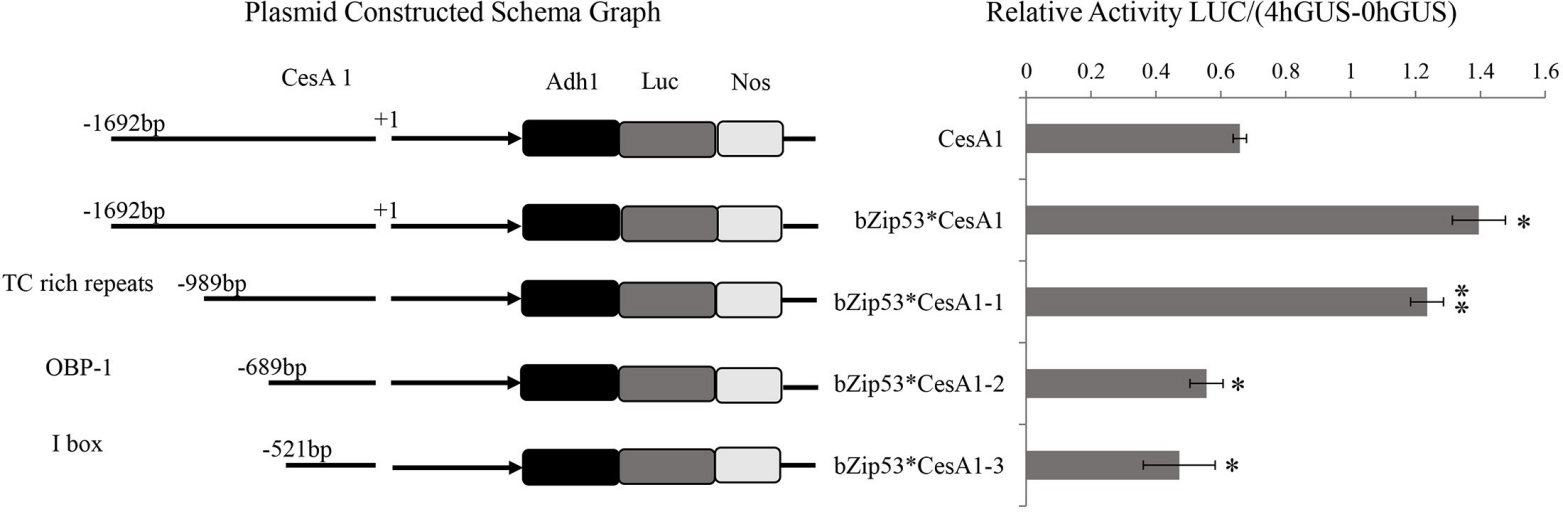
Preliminary determination of the binding region of *CesA1* promoter with *bZIP53*.

To further investigate the binding region of the TF *bZIP53* and the *CesA1* gene promoter, corresponding deletion fragments were constructed. Promoter analysis was performed on the *CesA1* gene promoter using PlantCare:(http://bioinformatics.psb.ugent.be/webtools/plantcare/html/), and the corresponding deletion vector was constructed. Twenty-four elements were predicted in the *CesA1* promoter, and the vectors after TC rich, OBP1, and I Box regions were selected after screening to construct the deletion elements named *CesA1*-1, *CesA1*-2, and *CesA1*-3, respectively. The effect of *bZIP53* on the activity of the promoter after deletion was verified by bombardment with a gene gun. As shown, the activity between bZIP53*CesA1-1 and bZIP53*CesA1-2 was significantly reduced, and the binding region of *bZIP53* to the *CesA1* gene promoter was determined to be between −989 bp and −689 bp (Fig 7). There were also five elements between −989 bp and −689 bp, namely ARE, GAG-motif, TCA-element, TGA-element, and Sp1, and the specific binding sites need to be further determined (Table 1).

**Table 1:** Elements in the biding region of *CesA1* promoter with the transcription factors *bZIP53*.

Motif No.	Motif name	Core sequence	Function
1	ARE	TGGTTT	Cis-acting regulatory element essential for the anaerobic induction
2	GAG-motif	GGAGATG	Part of a light responsive element
3	Sp1	CC(G/A)CCCA	Light responsive element
4	TCA-element	CCATCTTTTT	Cis-acting element involved in salicylic acid responsiveness
5	TGA-element	AACGAC	Auxin-responsie element

### Determination of *bZIP35* transcriptional activity

To test whether *bZIP53* has transcriptional activity, *bZIP53*-pGBKT was transferred into the yeast strain, and it was observed that the transformed yeast strain could grow normally on SD/-Trp medium, and *bZIP53*-pGBKT7 could grow normally on SD/-Trp/-His medium containing X-α-gal, but could not degrade the substrate blue, indicating that the protein encoded by the *bZIP53* gene could not activate the expression of the downstream His gene. The α-galactosidase degradation substrate is blue. The factor *bZIP53* does not have transcriptional activation activity and is unable to activate the expression of the reporter gene (S2 Fig).

#### Analysis of the transcription factor *bZIP53* by yeast one-hybrid assay

**Fig 8:**
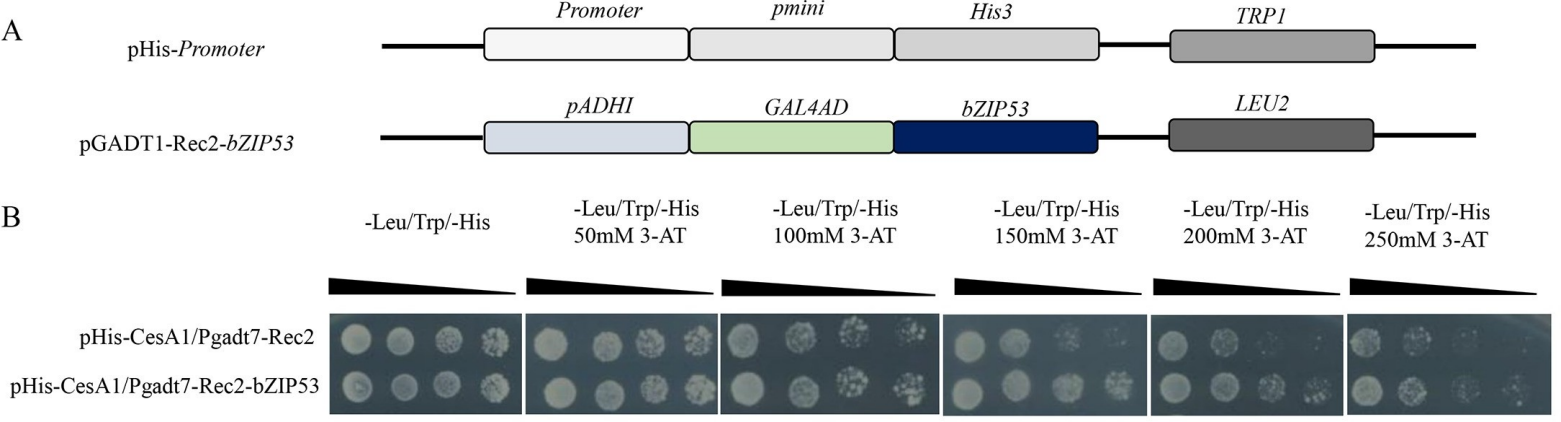
Identification of CesA1 regulation by *bZIP53* using yeast one hybrid assay. (A)Schematic representation of yeast expression construct; pGADT7-Rec2-bZIP53 and reporter construct; pHis2-CesA1 (CesA1 promoter). (B)Growth at different concentrations of 3-AT-deficient medium. SD/-Leu/-Trp/ His.pHis2-CesA1/pGADT7-Rec2 were used as negative controls.

The in vitro analysis of the TF *bZIP53* and the *CesA1* gene promoter was directly analyzed using the yeast one-hybrid assay. The yeast expression vector pGADT7-Rec2-*bZIP53* and pHis2-*CesA1* were constructed. The yeast expression vector was transformed into yeast Y187 strain by the LiAc-PEG method. pGADT7-Rec2-*bZIP53* was transformed with pHis2-*CesA1* into the experimental group, and pGADT7-Rec2 and pHis2-*CesA1* were co-transformed into the control group. After numerous experiments, it was found that the highest concentration of 3-AT in the laboratory did not inhibit the growth of yeast in the control group. All co-transformed yeasts were able to grow on the SD/-Leu/-Trp-deficient medium, and the growth was different at different concentrations of 3-AT-deficient medium SD/-Leu/-Trp/-His. The highest concentration of 3-AT did not inhibit the growth of the transformed yeast, but it was observed that the combination of the *CesA1* gene promoter and the TF *bZIP53* could grow at low 3-AT concentrations, but that high concentrations of 3-AT inhibited the growth. These interactions indicated a direct binding between the TF *bZIP53* and the *CesA1* gene promoter (Fig 8).

## Discussion

In the field experiment, the grain length and bulk density of maize seeds increased significantly after GA treatment. The results showed that the cellulose content of maize grain increased in different stages of grain maturity after GA treatment. The weight of a single seed was increased by up to 8% when treated with different concentrations of GA [31]. When corn plants were treated with different concentrations of GA, the 100-grain weight was increased to varying degrees [1]. GA can regulate the transcription of cellulose synthase and, thus, the synthesis of cellulose in the epidermis of rice vascular bundles [32]. The color of cellulose staining in the seed coat structure was deepened as the cellulose content increased. Compared with the control group, the grain endosperm was also observed to have changes in cell structure and the grain structure changed significantly at 25 DAP stage, but the extent of the influence on late grain development and size due to these two components was not clear. The observation of the seed coat showed that eight layers of cells could be observed at 15 DAP, the outer three layers of cells were the smallest, the density was high, and the degree of fibrosis and lignification of the seed coat cell walls gradually increased. The cell wall of the 20 DAP 4–8 layer was thickened, and the endosperm cells were visible. The 25 DAP showed that the contents in the pericarp cells disappeared, the cells squeezed and deformed each other due to dehydration, cells were highly lignified, and the thickness of pericarp decreased [31]. This was consistent with the results of corn grain sections observed in our study. Additionally, it was found that the degree of lignification of the GA-treated seed coat was lower than that of the untreated maize seed coat, which led to weakening from dehydration and reduced extrusion ability. In present study, the seed coat of the GA treatment became thinner, and the seed germination ability of the GA treatment group was greater than that of the control group.

In plants, basic region/leucine zipper motif (bZIP) transcription factors regulate processes including pathogen defence, light and stress signalling, seed maturation and flower development [33]. The TF *bZIP53* has the typical conserved domain of the *bZIP* family, the encoded protein is located in the nucleus, which can be detected in corn kernels, and the highest expression was found in 50 mM GA-treated kernels at 12 h. The yeast single hybrid transcription experiment showed that the TF *bZIP53* could interact with the *CesA1* gene promoters to increase their activity significantly. It was confirmed that the binding region of *bZIP53* to *CesA1* was between −989 bp and −689 bp, but the TF *bZIP53* itself was not activated. TF *bZIP53* could interact with the *CesA1* gene promoter, but it did not show transcriptional activation activity. Some studies on the TF *bZIP53* have shown that *bZIP53* alone, driven by Pro35s, cannot affect the activity of the GUS reporter gene in Arabidopsis thaliana leaves [34]. It is speculated that the effect of the transcription factor *bZIP53* on the *CesA1* gene promoter is similar. The factors interacting with transcription factor *bZIP53* should be further verified by further research.

In the experiment validating the promoter of the transcription factor and cellulose synthase gene by yeast one hybrid assay, the growth of the control group was still not significantly inhibited in the medium with 300 mM 3-AT. It was found that the chemical composition of the yeast cell wall is unique, and is mainly composed of "yeast cellulose." It is similar to a sandwich, mannan on the outer layer, dextran in the inner layer, and a layer of protein molecules forming the outer layer. Yeast needs similar nutrients as other living organisms, such as bacteria, and it possesses a set of intracellular and extracellular enzyme systems that are used to decompose macromolecular substances into smaller molecular substances that are easily used in cell metabolism. There is some conjecture as to whether or not cellulose can be decomposed in the process of yeast growth so that the substances needed for its growth can be synthesized, resulting in its ability to grow under the inhibition of a high concentration of 3-AT, but this inference needs to be verified.

## Supporting information

S1 Table Predicted components of the *CesA1* promoter. (TIF)

S2 Table Family and number of transcription factors screened after GA treatment. (TIF)

S3 Table Target gene and log value from screening. (TIF)

S4 Table Co-correlation analysis between transcription factor *bZIP53* and cellulose synthase regulated by GA in RNA-Seq. (TIF)

S1 Fig *bZIP53* amplification. (TIF)

S2 Fig Transcriptional activation activity of the *bZIP53*. pGBKT7-GAL4 AD was the positive control. pGBKT7 and bZIP35-pGBKT7 were the negative controls.
(TIF)

## Abbreviations

GA: gibberellin
CesA1: cellulose synthase gene 1
bZIP53: basic region/leucine zipper motif 53
TF: transcription factor
DAP: days after pollination
