# Supplementary figures and images for: Gibberellin induced transcription factor bZIP53 regulates CesA1 expression in maize kernels
Source: PLoS One. 2021 Mar 17;16(3):e0244591. doi: 10.1371/journal.pone.0244591 (PMC7968625; doi:10.1371/journal.pone.0244591)

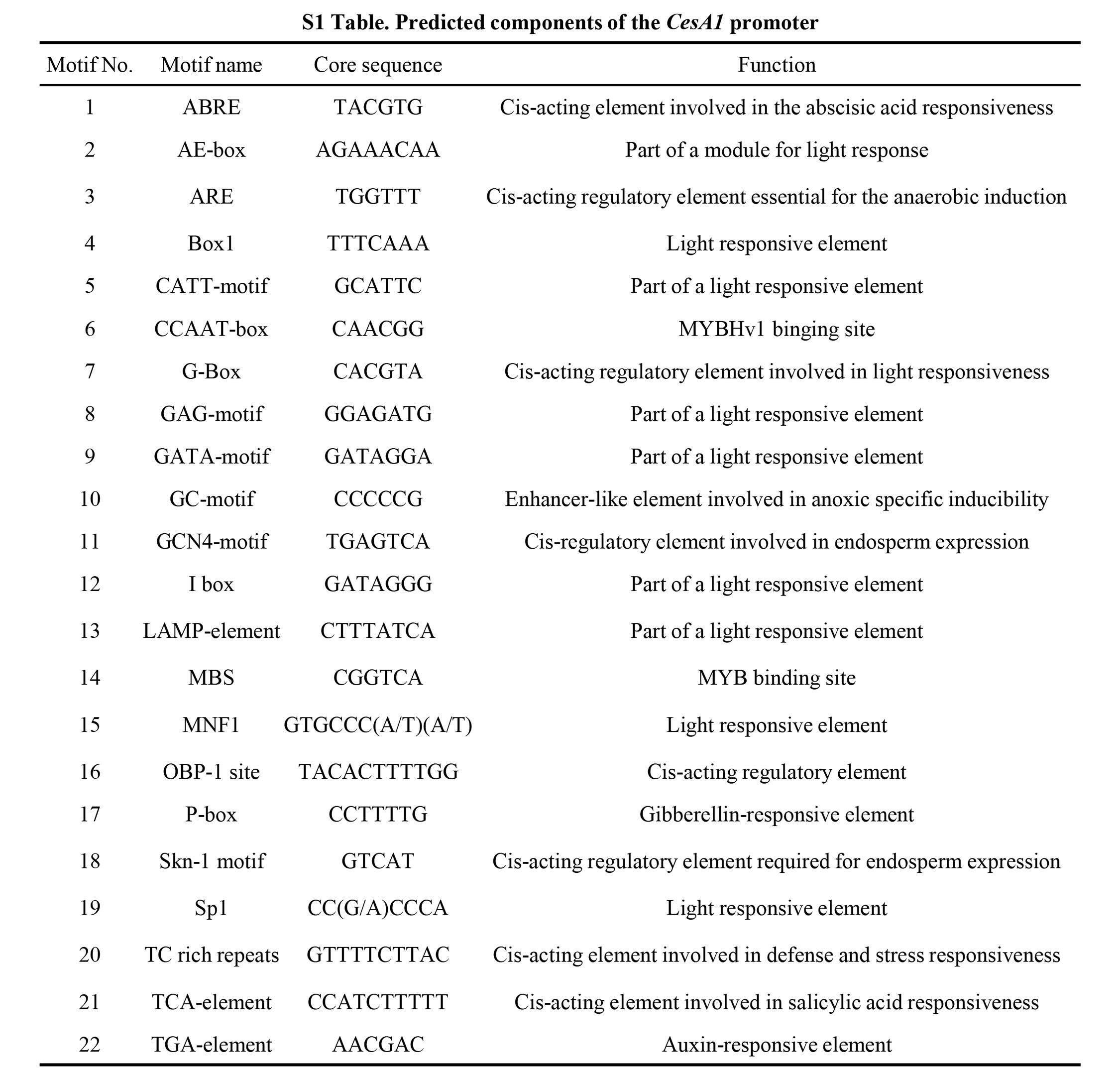

Supplement: S1 Table — (TIF) [file pone.0244591.s001.tif]

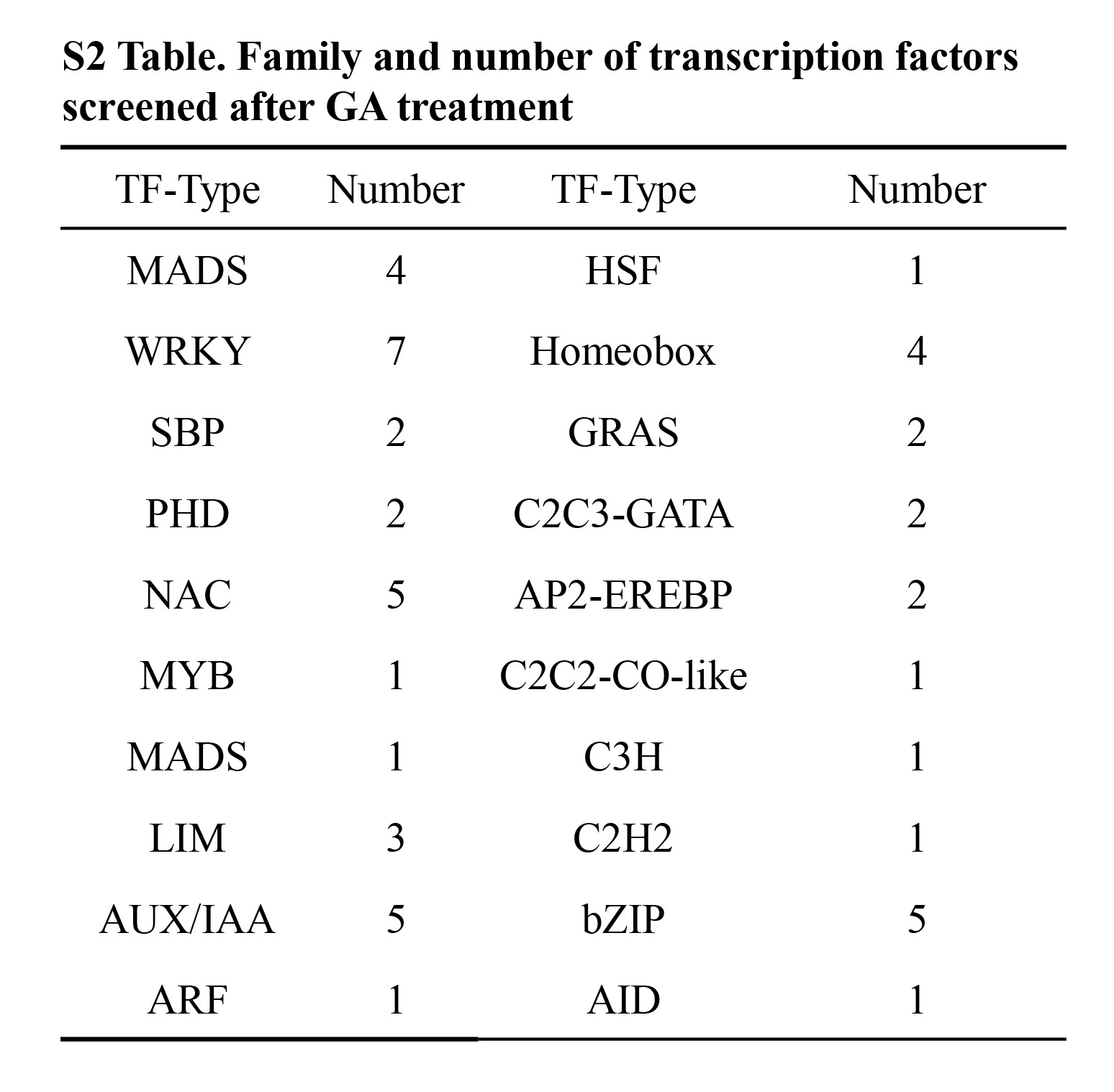

Supplement: S2 Table — (TIF) [file pone.0244591.s002.tif]

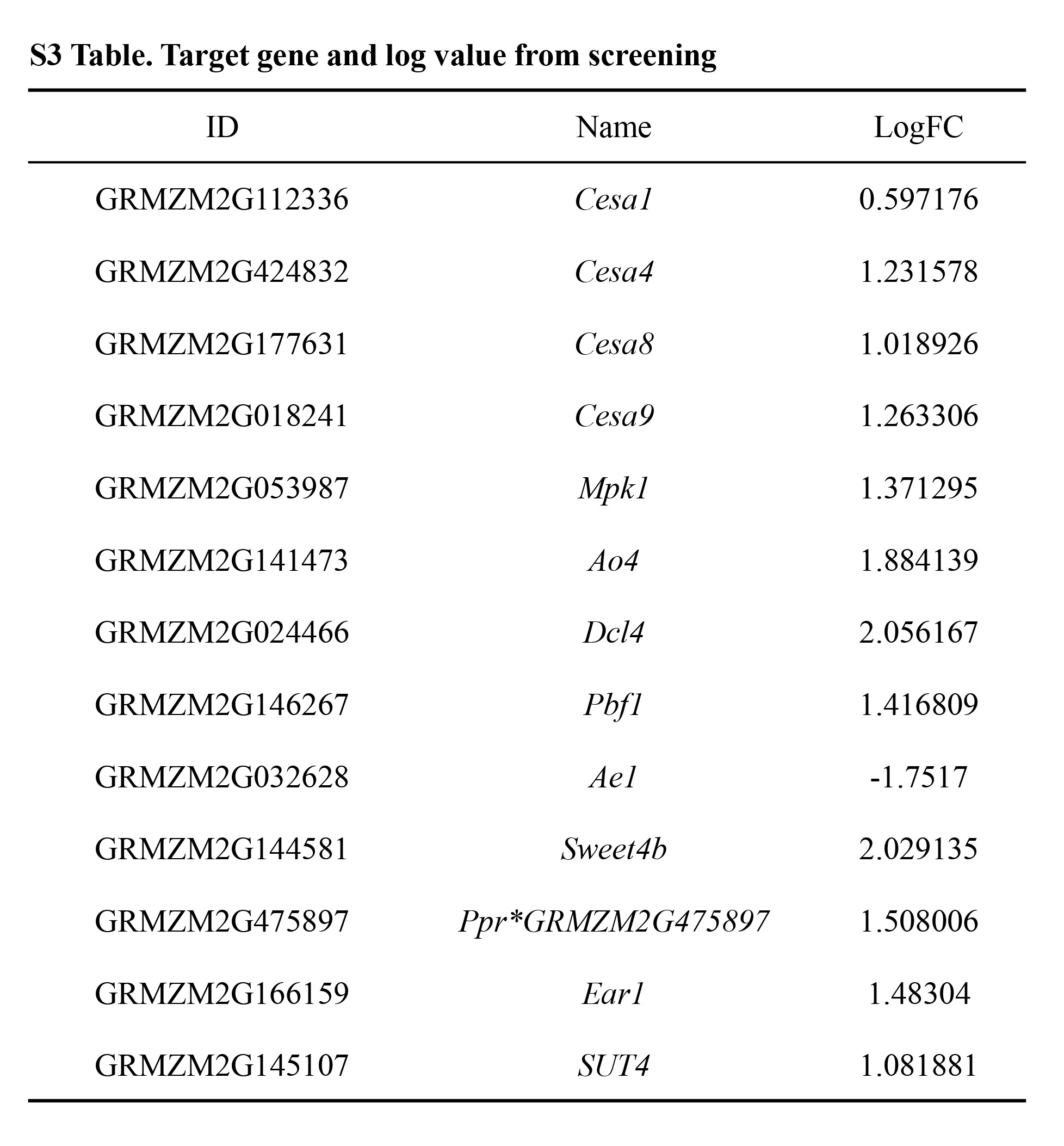

Supplement: S3 Table — (TIF) [file pone.0244591.s003.tif]

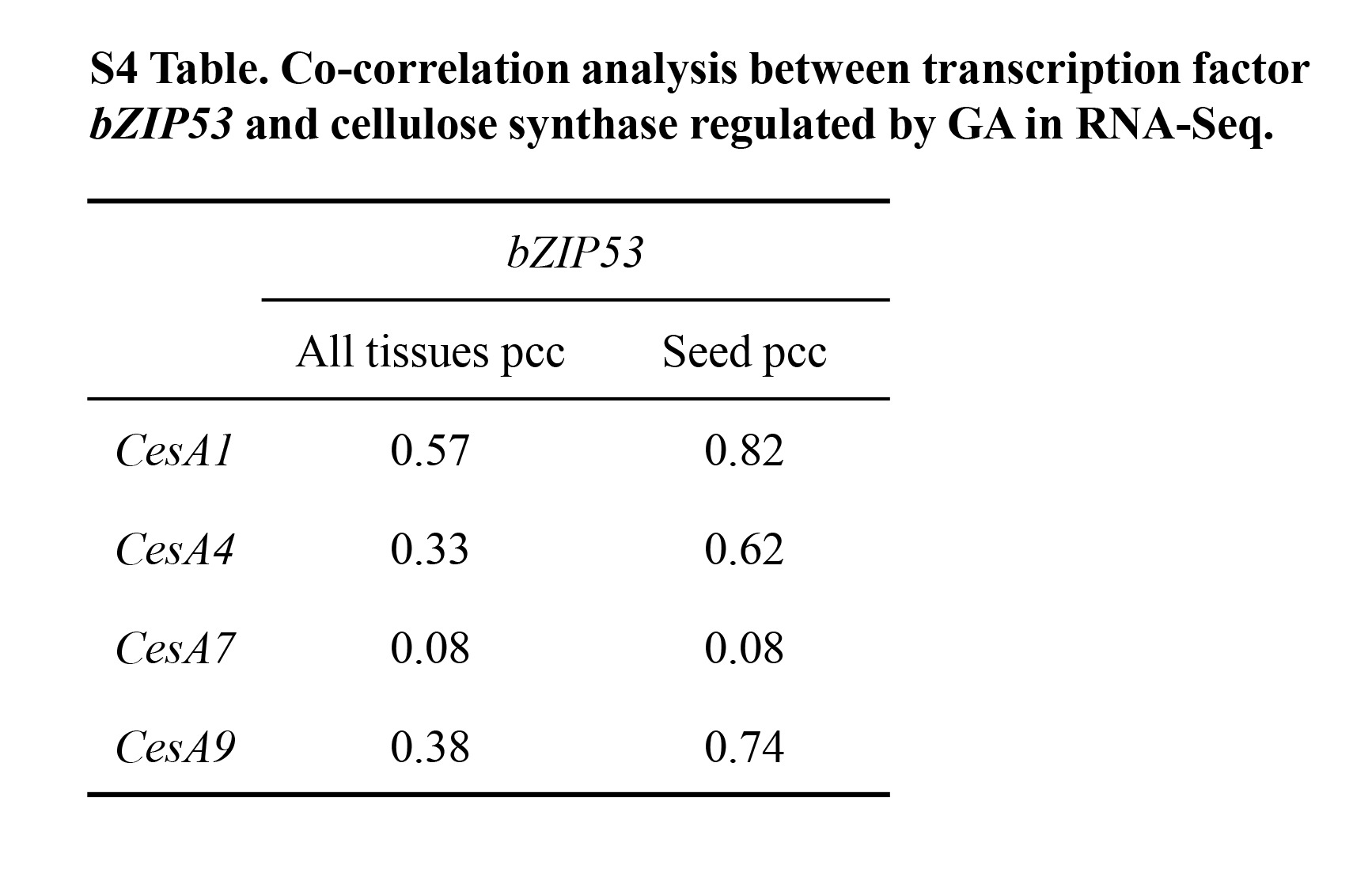

Supplement: S4 Table — (TIF) [file pone.0244591.s004.tif]

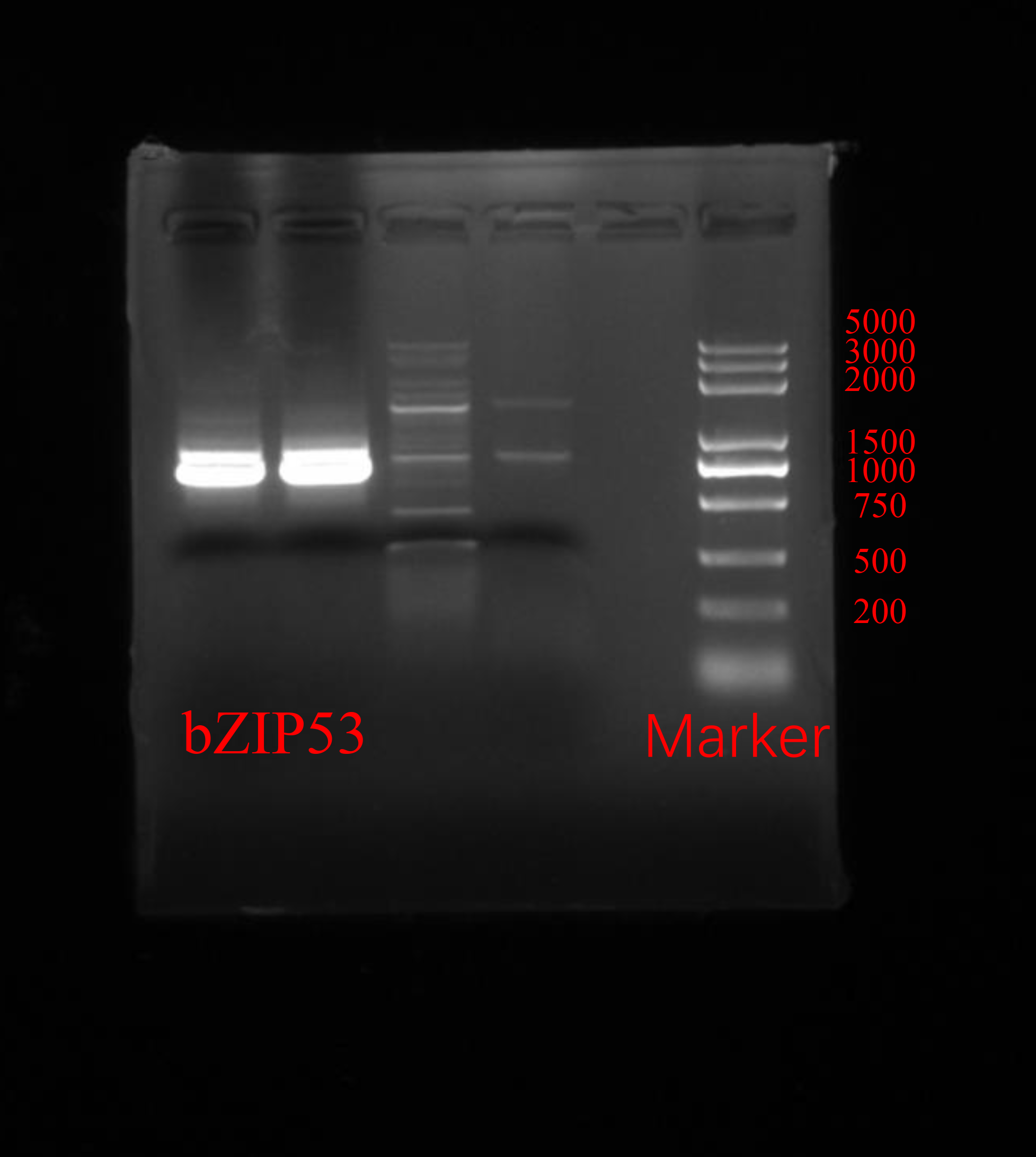

Supplement: S1 Fig — (TIF) [file pone.0244591.s005.tif]

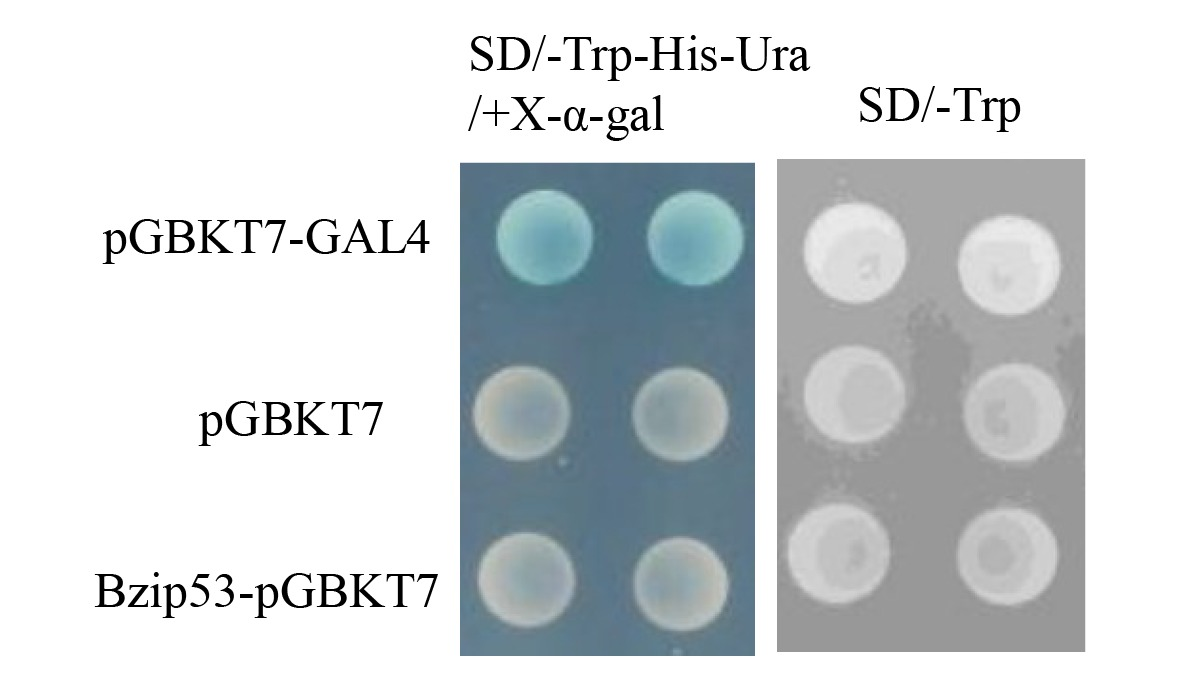

Supplement: S2 Fig — pGBKT7-GAL4 AD was the positive control. pGBKT7 and bZIP35-pGBKT7 were the negative controls. (TIF) [file pone.0244591.s006.tif]
